# Differentiation between wild and artificial cultivated *Stephaniae tetrandrae* radix using chromatographic and flow‐injection mass spectrometric fingerprints with the aid of principal component analysis

**DOI:** 10.1002/fsn3.1717

**Published:** 2020-06-23

**Authors:** Ya‐dong Qin, Feng‐man Fang, Rong‐bin Wang, Juan‐juan Zhou, Lin‐hua Li

**Affiliations:** ^1^ College of Life Sciences Anhui Normal University Wuhu China; ^2^ Pharmacy Department Anhui College of Traditional Chinese Medicine Wuhu China; ^3^ Pharmacy Division Wuhu Hospital of Traditional Chinese Medicine Wuhu China

**Keywords:** chromatographic, flow‐injection mass spectrometric, principal component analysis, *Stephaniae tetrandrae* radix

## Abstract

High‐performance liquid chromatographic (HPLC) and flow‐injection mass spectrometric (FIMS) fingerprinting profiles were used to differentiate between wild and artificial cultivated *Stephaniae tetrandrae* Radix samples. HPLC and FIMS fingerprints of 15 wild *S. tetrandrae* Radix samples and 12 artificial cultivated *S. tetrandrae* Radix samples were obtained and analyzed with the aid of principal component analysis (PCA). PCA of the fingerprints showed that the chemical differences between wild and artificial cultivated *S. tetrandrae* Radix samples could be differentiated by either HPLC or FIMS fingerprints. The HPLC fingerprints provided more chemical information but required longer analytical time compared with FIMS fingerprints. This study indicated that the wild samples contained higher concentrations of almost all of the major compounds than the cultivated samples. Three characteristic compounds which were responsible for the differences between the samples were tentatively identified with the aid of MS data. Furthermore, these three compounds, tetrandrine (TET), fangchinoline (FAN), and cyclanoline (CYC), were quantified. The HPLC and FIMS fingerprints combined with PCA could be used for quality assessment of wild and artificial cultivated *S. tetrandrae* Radix samples.

## Graphical abstract

Principal component analysis score plot (above) of FIMS fingerprints displays obviously that wild (WS) and artificial cultivated (CS) *Stephaniae tetrandrae* Radix samples are clearly separated from each other by the first principal component (PC1). WS samples are mainly clustered on the left in the negative PC1 area, with PC1 scores below zero. However, CS samples are gathered to the right side in the positive PC1 area, with PC1 scores above zero. According to PCA loadings plot (below) of FIMS fingerprints, the ions at m/z 609, 623, and 343 contribute negatively to PC1. The effects of absolute intensity values of these ions decide the position of each sample. These three ions were the most important for differentiation between WS and CS samples. They were identified as tetrandrine, fangchinoline, and cyclanoline, respectively, which were then quantitated due to the availability of the commercial reference compounds.

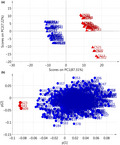

## 1 INTRODUCTION

The dried root of *Stephania tetrandrae* S. Moore, Fangji in Chinese, has been widely used clinically for more than 2,000 years and shown significant diuretic and antirheumatic effects (Bhagya & Chandrashekar, 2016; Yu, Wu, Chen, Pang, & Wong, 2004). Its main compounds are alkaloids, including tetrandrine (TET), fangchinoline (FAN) et al., well‐known to act as a calcium channel blocker and wide range of pharmacological activity. Besides modern pharmacology research, some other studies indicated that TET and FAN could regulate nutrients and mineral elements level, such as TET has significant effect on glucose metabolism (Sun et al., 1992). TET and FAN have a weak inhibitory effect on protein synthesis (Liu et al., 1990). FAN could decrease copper levels while it increased zinc content (Lu, Liu, Li, & Fan, 2019). These results reveal the beneficial health effects are closely related to TET, FAN, and alkaloids compounds.

Historically, the dried root of *Aristolochia fangchi* Y.C.Wu ex L.D. Chou et S.M. Hwang was also used as Fangji in China. Since the late twentieth century, *A. fangchi* has been found to be nephrotoxic and carcinogenic due to containing aristolochic acids (Arlt, Stiborova, & Schmeiser, 2002; Rietjens, Martena, Boersma, Spiegelenberg, & Alink, 2005; Wojcikowski, Johnson, & Gobe, 2004). Therefore, since 2005, *S. tetrandrae* Radix has been officially specified as authentic medicinal herb, whereas *A. fangchi* has been considered as its counterpart.

At present, almost all the *S. tetrandrae* Radix medicinal plants are wild. With the increasing demand of the herb, the plants were over collected, causing acute shortage of the herb (Huang, Liu, & Li, 2008; Qing & Wang, 2007). A few enterprises and medicine material farms are trying to cultivate *S. tetrandrae* S. Moore. However, *S. tetrandrae* Radix cannot be applied in large scale for lack of cultivation techniques. To the best of my knowledge, the quality assessment on artificial cultivated *S. tetrandrae* Radix has never been reported.

High‐performance liquid chromatographic (HPLC) fingerprints (Li, Zhang, & Yang, 2017; Liang et al., 2019; Zhou, Qin, Wang, & Huang, 2018) and determination, identification of active chemical components (Liu et al., 2018; Lu et al., 2015; Sim, Kim, Lee, & Hong, 2013; Wang et al., 2015) of *S. tetrandrae* Radix and its prescription were reported. However, no study has been reported on comparison between wild and cultivated *S. tetrandrae* Radix. In our previous studies, chromatographic fingerprints combined with chemometrics have been successfully used to evaluate the quality of *S. tetrandrae* Radix collected from different habitats (Zhou et al., 2018). On the top of this, we aimed to differentiate between wild and cultivated *S. tetrandrae* Radix using chromatographic and FIMS fingerprints combined with chemometrics.

## 2 MATERIALS AND METHODS

### 2.1 Samples

Fifteen batches of wild *S. tetrandrae* Radix samples (WS) and twelve batches of cultivated *S. tetrandrae* Radix samples (CS) were collected from different habitats in south of Anhui province and north of Jiangxi province, which were the main production area of *S. tetrandrae* Radix in China (listed in Table 1). The cultivated *S. tetrandrae* herb was planted in April 2013, and the growing year of wild and artificial *S. tetrandrae* herb was about 4 years. All the samples were dried in shade and authenticated by Professor Rongbin Wang from Anhui Institute of Traditional Chinese Medicine Resources.

**TABLE 1:** The detailed information of the tested samples and the concentration values (mg/g) of the three alkaloids in these samples

Label	Locality	Source	Collection date	TET	FAN	CYC
WS1	Huangshan, Anhui	Wild	Oct 2017	16.53 ± 0.12	8.75 ± 0.06	0.21 ± 0.05
WS2	Shitai, Anhui	Wild	Oct 2017	12.11 ± 0.14	9.22 ± 0.05	0.34 ± 0.03
WS3	Chizhou, Anhui	Wild	Nov 2017	15.32 ± 0.07	7.51 ± 0.02	0.26 ± 0.03
WS4	Xiushui, Jiangxi	Wild	Oct 2017	14.17 ± 0.11	6.57 ± 0.02	0.24 ± 0.03
WS5	Yichun, Jiangxi	Wild	Oct 2017	9.35 ± 0.03	4.91 ± 0.01	0.27 ± 0.01
WS6	Wuhu, Anhui	Wild	Oct 2017	11.57 ± 0.13	5.68 ± 0.01	0.22 ± 0.01
WS7	Fuliang, Jiangxi	Wild	Nov 2017	12.52 ± 0.12	6.08 ± 0.02	0.25 ± 0.01
WS8	Zhangjiajie, Hunan	Wild	Dec 2017	10.72 ± 0.12	4.51 ± 0.01	0.36 ± 0.00
WS9	Ningguo, Anhui	Wild	Nov 2017	7.24 ± 0.02	7.33 ± 0.02	0.20 ± 0.01
WS10	Shangrao, Jiangxi	Wild	Oct 2017	6.57 ± 0.02	5.68 ± 0.01	0.31 ± 0.02
WS11	Xuancheng, Anhui	Wild	Oct 2017	7.14 ± 0.03	5.02 ± 0.01	0.26 ± 0.01
WS12	Suichuan, Jiangxi	Wild	Oct 2017	8.25 ± 0.06	8.51 ± 0.07	0.17 ± 0.00
WS13	Qimen, Anhui	Wild	Nov 2017	8.66 ± 0.05	6.57 ± 0.02	0.37 ± 0.02
WS14	Chizhou, Anhui	Wild	Nov 2017	7.59 ± 0.06	7.95 ± 0.02	0.29 ± 0.02
WS15	Yichun, Jiangxi	Wild	Nov 2017	9.12 ± 0.09	7.74 ± 0.01	0.33 ± 0.04
CS1	Huangshan, Anhui	Cultivated	Oct 2017	4.35 ± 0.00	2.31 ± 0.00	0.11 ± 0.00
CS2	Gaoan, Jiangxi	Cultivated	Dec 2017	5.21 ± 0.00	3.12 ± 0.00	0.05 ± 0.00
CS3	Shangrao, Jiangxi	Cultivated	Oct 2017	4.21 ± 0.00	2.74 ± 0.00	0.11 ± 0.02
CS4	Shitai, Anhui	Cultivated	Oct 2017	4.45 ± 0.00	3.71 ± 0.00	0.06 ± 0.00
CS5	Yuexi, Anhui	Cultivated	Nov 2017	6.09 ± 0.01	2.16 ± 0.00	0.08 ± 0.00
CS6	Huangshan, Anhui	Cultivated	Oct 2017	6.26 ± 0.01	2.15 ± 0.00	0.07 ± 0.00
CS7	Zhuzhou, Hunan	Cultivated	Oct 2017	5.24 ± 0.00	3.91 ± 0.01	0.09 ± 0.01
CS8	Suichuan, Jiangxi	Cultivated	Nov 2017	6.27 ± 0.01	4.36 ± 0.01	0.08 ± 0.00
CS9	Pengze, Jiangxi	Cultivated	Nov 2017	6.12 ± 0.03	3.28 ± 0.01	0.11 ± 0.00
CS10	Yichun, Jiangxi	Cultivated	Oct 2017	5.84 ± 0.02	4.02 ± 0.01	0.10 ± 0.00
CS11	Shangrao, Jiangxi	Cultivated	Oct 2017	6.38 ± 0.01	3.82 ± 0.00	0.12 ± 0.03
CS12	Taihe, Jiangxi	Cultivated	Nov 2017	5.44 ± 0.04	3.47 ± 0.00	0.09 ± 0.00

Note

Date are expressed as mean ± *SD* of duplicate experiments

### 2.2 Reference standards

Reference standards of fangchinoline (FAN) and tetrandrine (TET) were acquired from National Institutes for Food and Drug Control. The reference standard of cyclanoline (CYC) and magnoflorine (MAG) was obtained from Chengdu Must Bio‐technology Co., LTD and Shanghai Yuanmu Bio‐technology Co., LTD, respectively. Their purities were above 98% by HPLC analysis.

### 2.3 Reagents

Acetonitrile (Tedia) and formic acid (Aladdin) were MS grade. Water was Wahaha pure water (Wahaha Food and Beverage Ltd.). All other regents were of analytical grade.

### 2.4 Sample preparation

The *S. tetrandrae* Radix samples were dried and ground to power at 60‐mesh particle size and stored at −4°C prior to analysis. Two hundred milligrams of each sample was weighed and extracted with 20 ml of methanol:water (50/50, v/v) in a 25‐ml centrifuge tube by sonication at room temperature for 60 min. The extracts were filtered through 0.22 μm Nylon syringe filter (Xinya purification equipment) for analysis within 24 hr after extraction. Each extract was analyzed three times. The concentration was 10 mg/ml for HPLC analysis. Each sample solution was diluted five times with methanol–water (50/50, v/v) to obtain the concentration at 2 mg/ml prior to FIMS analysis. Each extract was analyzed three times.

All the samples were collected with satisfied sample sizes and fully represent the genuine WS and CS *S. tetrandrae* Radix samples.

### 2.5 HPLC and FIMS Conditions

The HPLC analysis was performed according to our previously reported laboratory procedure (Zhou et al., 2018). The Agilent 1,290 UHPLC system consists of quadruple pump, online degasser, auto‐sampler, a diode‐array detector, and controlled column compartment. The LC‐MS analysis was performed on Triple Quadrupole (SHIMADZU 8030) mass spectrometer connected to Shimadzu LC binary high‐pressure gradient HPLC system.

The chromatographic separation of samples was carried out on an Agilent Eclipse plus C18 column (150 × 4.6 mm, 3.5 μm particle size) from Agilent Technologies. The binary UHPLC elution mobile phase consisted of 0.2% formic acid in acetonitrile (A) and 0.2% formic acid in water (B). The mobile phase flow rate was maintained at 0.7 ml/min. The gradient program was as follow: 0–10 min, 15% A to 30% A; 10–15 min, 30% to 40% A; 15–25 min, 40% to 60% A. The column temperature was maintained at 25°C and injection volume of 2 μl. The DAD wavelength was set to 270 nm. Three injections were performed for each sample.

The FIMS fingerprint for each sample was achieved by an Agilent SB C18 guard column (5 × 2.1 mm, 1.8 μm particle size) from Agilent Technologies. The mobile phase consisted of 0.2% formic acid in acetonitrile (A) and 0.2% formic acid in water (B). The ratio between A and B was 50:50 for 2 min with a flow rate of 0.3 ml/min. The column temperature was maintained at 25°C. Sample solution was diluted five times with methanol–water (50/50, v/v), and the injection volume was 2 μl. The following electrospray ionization (ESI) in positive ion mode parameters was optimized and used: sheath gas flow rate 80 arb; aux gas flow rate 10 arb; spray voltage, 3.0 kV; heated capillary temperature, 250°C; capillary voltage, −4.0 V; and tube lens offset, 20 V. The MS spectra were collected from 0.1 to 1.1 min, and the mass range was from 100 to 1,000 m*/z*. Triplicate analyses of the 27 *S. tetrandrae* Radix samples provided 81 MS spectra.

### 2.6 Data analysis

Data analysis was performed using Similarity Evaluation System (SES) for Chromatographic Fingerprint of Traditional Chinese Medicine software (Version 2004A, Chinese Pharmacopoeia Committee), which was recommended by the State Food and Drug Administration of China (SFDA). To get rid of the solvent interferences, the chromatographic data used for peak integration were retention time between 2 and 20 min. Eight peaks from each chromatographic fingerprint were selected as common peaks. Then, the absolute peak area was exported from SES and saved in.csv format. Peak 7, the predominant peak in both the WS and CS samples, was selected as the reference peak. Data sets of absolute peak areas of the eight common peaks and the relative ratios of the other seven peaks to the reference peak were both used for PCA.

Differences between means were calculated by analysis of variance (ANOVA) with Tukey's HSD post hoc test, utilizing IBM SPSS Statistics 22.0 software (IBM). All data points from FIMS chromatogram of each sample were export from LabSolutions software and saved in.csv format. The FIMS fingerprint data consisted of *m/z* 100–1000. The 81 spectra were combined. The information that was not relevant to PCA was deleted. Then, intensities of all ions were imported into in Excel (Microsoft, Inc.). Next, each missing *m/z* in mass list was filled with zero so that each sample contained 901 data points. Finally, two‐dimensional matrix (81 samples × 901 masses) was then export to SIMCA‐P for PCA. Preprocessing in SIMCA‐P, prior to PCA, consisted of normalization and mean centering.

## 3 RESULTS AND DISCUSSION

### 3.1 Quantitative analysis

In this study, the contents of TET, FAN, and CYC in 27 batches of *S. tetrandrae* Radix samples were quantified. The concentration values of three compounds' mean and standard deviation (*SD*) are displayed in Table 1. The results showed that TET, FAN, and CYC are the three major alkaloids in the samples. The mean concentration values for TET, FAN, and CYC were 10.46, 6.80, and 0.27 mg/g in wild samples, respectively. The values were two times higher than that in cultivated samples (*p* < .01).

### 3.2 Chromatographic and FIMS fingerprints

**FIGURE 1:**
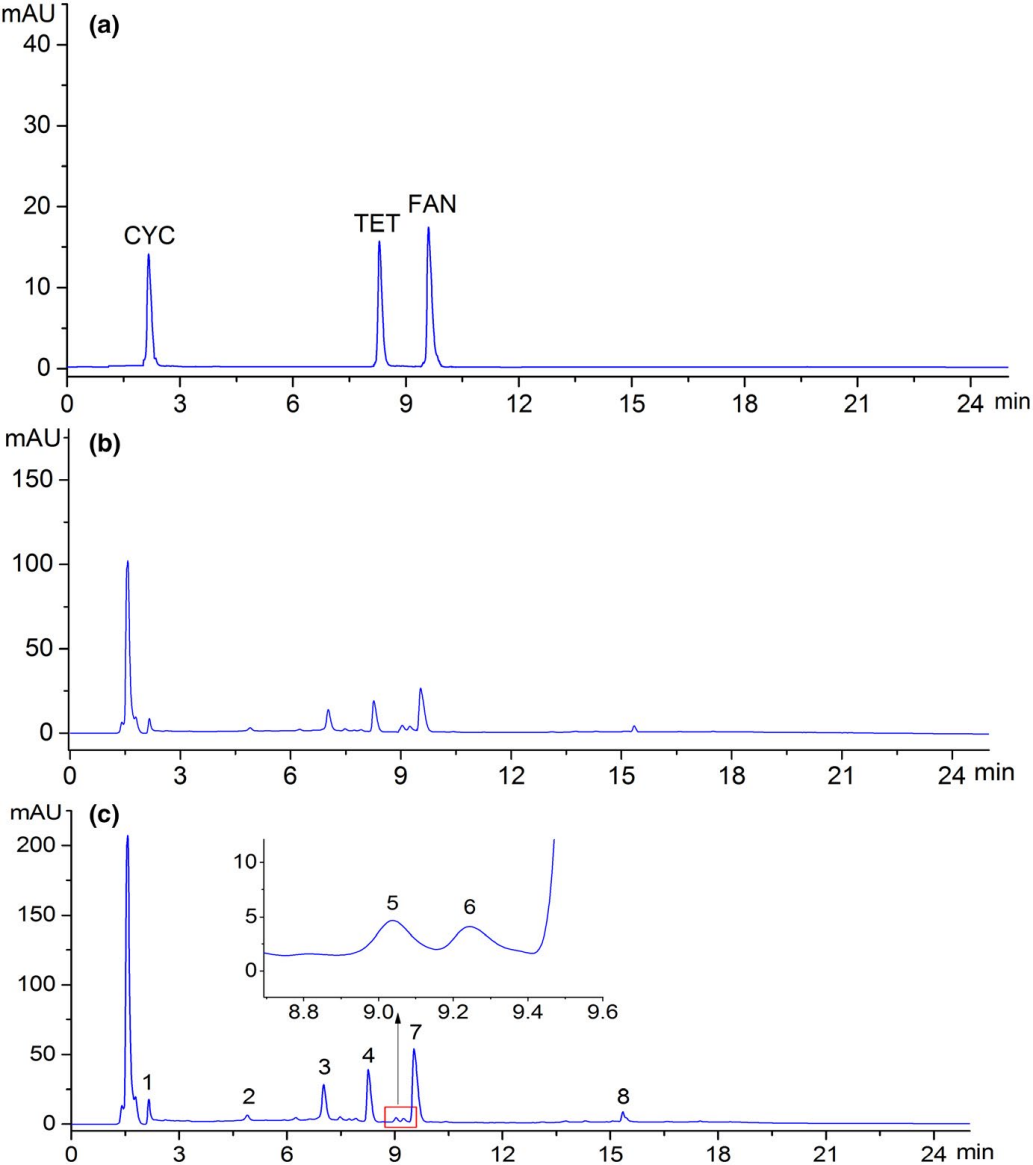
HPLC chromatograms of mixed standards and representative HPLC chromatograms of *Stephaniae tetrandrae* Radix samples: (a) mixed standards; (b) cultivated *S. tetrandrae* Radix sample; (c) wild *S. tetrandrae* Radix samples. Peaks 1, 2, 4, and 7 were CYC, MAG, TET, and FAN, respectively

Typical chromatographic fingerprints for mixed standards, wild, and cultivated samples are shown in Figure 1. A total of eight common peaks were visible and selected as the common peaks in the analysis of the 21 batches of samples by SES, and HPLC fingerprints of 27 batches samples were showed in Supplementary Figure A. The peaks of TET, FAN, MAG, and CYC were predominant and confirmed by commercially available reference compounds. Plus, other common peaks were tentatively identified by comparing their MS and UV data with that in previously published literatures (Table 2). The average peak area of TET, FAN, and CYC was 271, 493, and 127 in wild samples, which were about two times of that in cultivated samples, on weight equivalent basis. In addition, the peak area of other five common compounds was too small so that the average area of them was notably different between wild and cultivated samples.

**TABLE 2:** Identified components in tested *S. tetrandrae* Radix samples

Peak number	*t*_R_(min)	[M + H]^+^/[M^+^]	Compound	Reference
1	2.16	343.25	Cyclanoline	Reference standard (Liu et al., 2018)
2	4.94	342.40	Magnoflorine	Reference standard (Sim et al., 2013)
3	7.05	340.45	Nantenine	Literature (Liu et al., 2018)
4	8.31	609.35	Tetrandrine	Reference standard (Liu et al., 2018; Sim et al., 2013; Wang et al., 2015)
5	9.05	415.95	Unknown	/
6	9.29	453.50	Unknown	/
7	9.62	623.30	Fangchinoline	Reference standard (Liu et al., 2018; Sim et al., 2013; Wang et al., 2015)
8	15.36	368.45	Unknown	/

**FIGURE 2:**
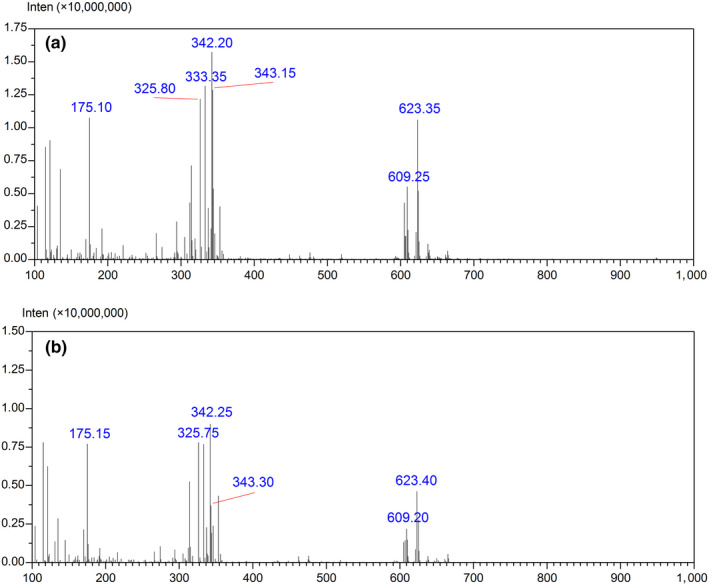
FIMS of wild (a) and cultivated (b) *Stephaniae tetrandrae* Radix samples

Typical FIMS fingerprints for the wild and cultivated *S. tetrandrae* Radix samples are shown in Figure 2. The most notable ions of both WS and CS samples are at *m/z* 175, 326, 342, 343, and 623. It can be seen that they have different intensities of these common ions. However, the specific differences need to be revealed with the aid of chemometrics.

### 3.3 PCA of chromatographic fingerprints

Principal component analysis concerns a mathematical procedure that transforms a number of possibly correlated variables into a smaller number of uncorrelated variables (Huang et al., 2020; Zhao, Chang, & Chen, 2015). PCA provides visual patterns (Castro‐Alba et al., 2019; Chen, Sun, & Ford, 2014; Reale et al., 2020; Sun, Sun, & Han, 2018) which can be understood and accepted easily; furthermore, the results also avoid subjective decisions. In the present study, to assess the resemblance and differences between wild and cultivated samples, a PCA was performed based on the eight common peaks (Figure 1). The absolute peak areas of the eight common peaks in the 27 chromatograms of *S. tetrandrae* Radix samples formed a matrix of 8 × 27. The data were mean‐centered before analysis.

**FIGURE 3:**
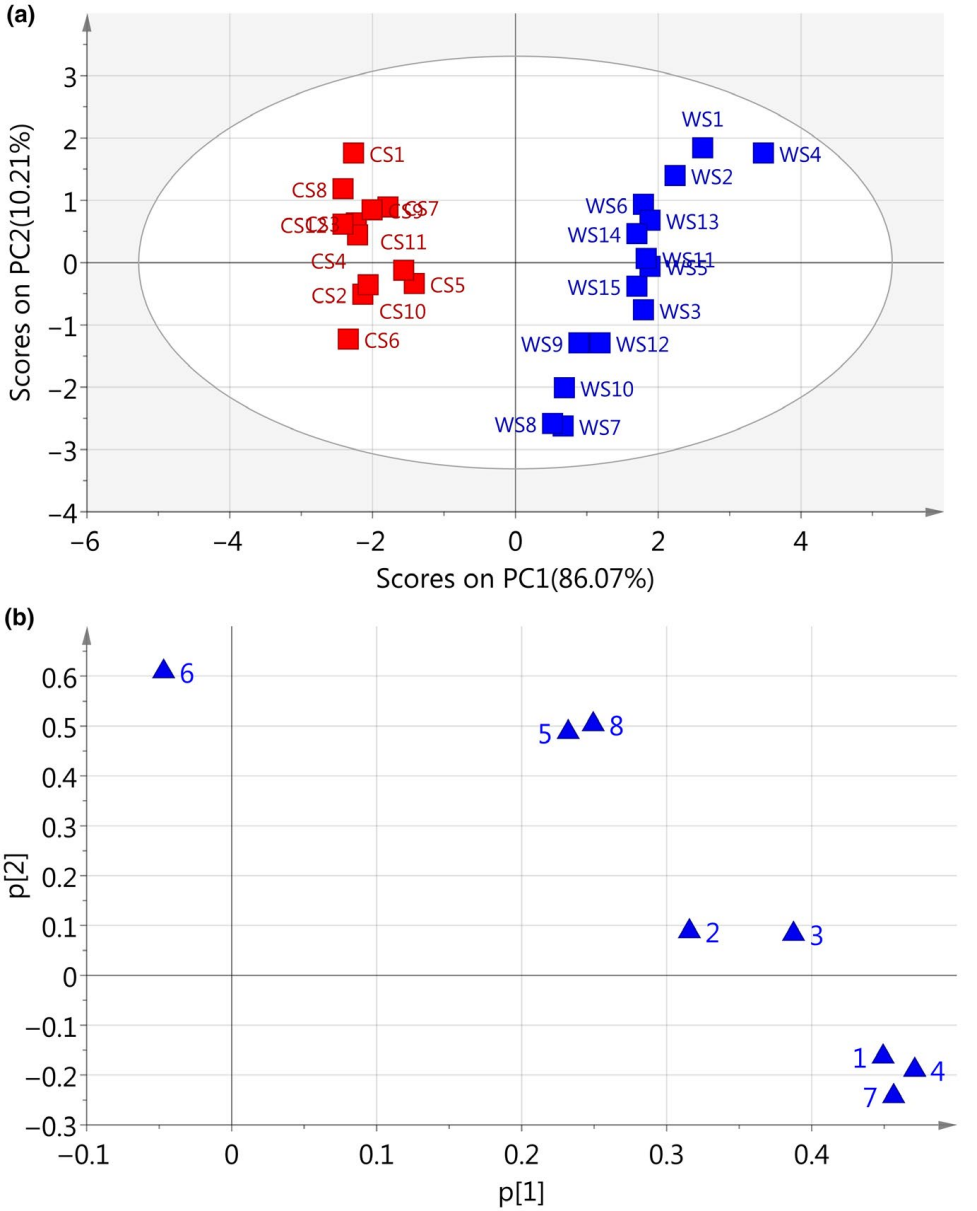
PCA scores plot (a) and loadings plot (b) for absolute peak areas in HPLC fingerprints of *Stephaniae tetrandrae* Radix samples

The scores plot and loadings plot of the PCA are plotted based on the chromatographic absolute peak areas of the eight common peaks. The PCA scores plot (Figure 3a) indicated that the wild *S. tetrandrae* samples (WS1‐WS15) were located in the right side in the positive PC1 area. They were discriminated against the cultivated *S. tetrandrae* samples which were located on the left side in the negative PC1 area. The PCA loadings plot (Figure 3b) displayed that CYC (peak 1), TET (peak 4) and FAN (peak 7) play the most important role in discriminating wild and cultivated *S. tetrandrae* samples. CYC, TET, and FAN contribute positively to PC1, as can be seen in PC1 loadings plot (Supplementary Figure B). We found that the contents of them were statistically significant (*p* < .01) in wild *S. tetrandrae* samples than that in cultivated *S. tetrandrae* samples.

**FIGURE 4:**
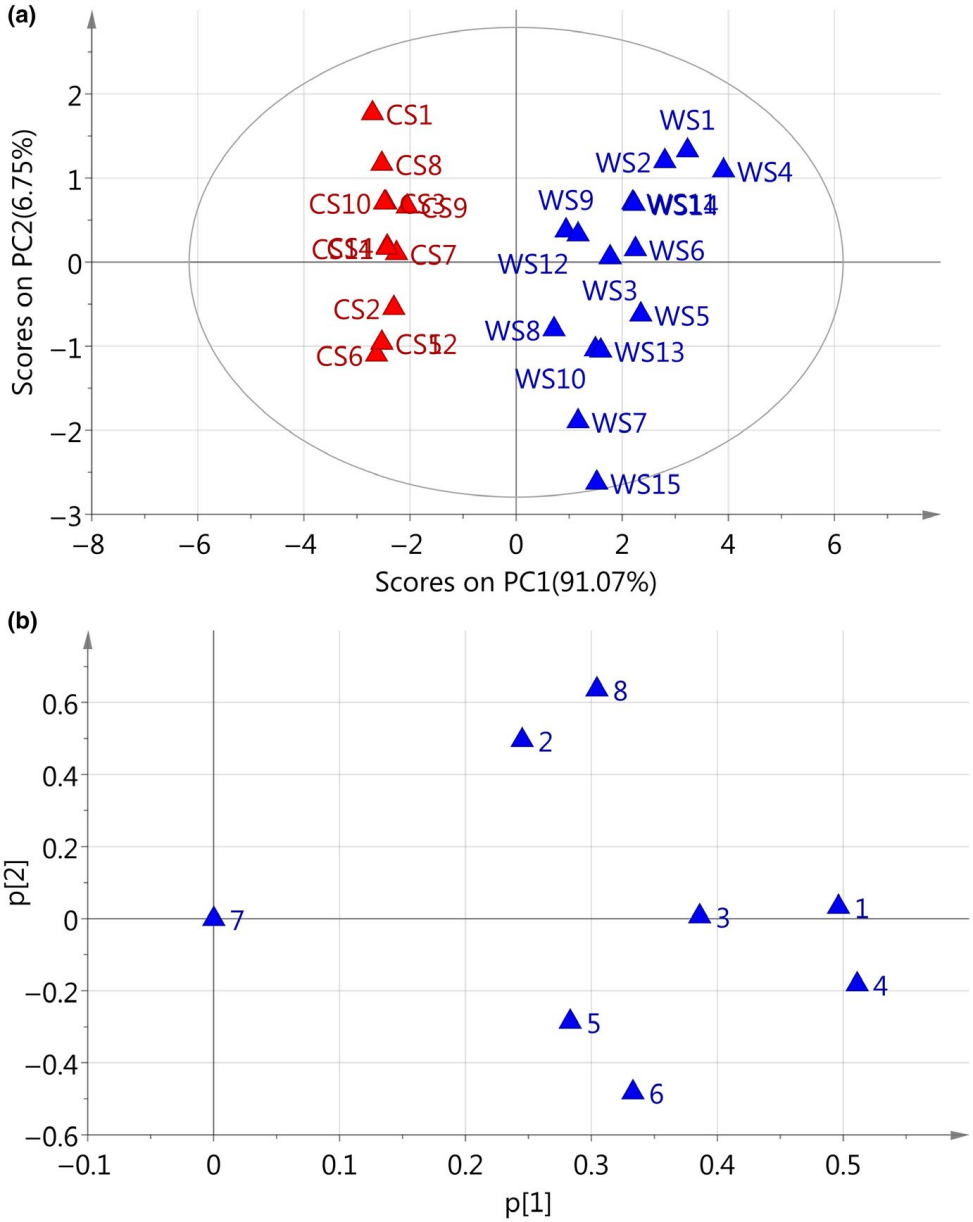
PCA scores plot (a) and loadings plot (b) for relative peak areas in HPLC fingerprints of *Stephaniae tetrandrae* Radix samples

Figure 4a,b display peak area‐based scores plot and loadings plot, respectively, of the WS and CS samples. From the scores plot, we can see that WS and CS samples are clearly separated into two groups. All the WS samples are clustered to the right of the plot, whereas all the CS samples are clustered to the left. The results were the same as what we obtained from peak area‐based PCA. PCA loadings plot (Figure 4b) illustrates that peak 1 (CYC) and 4 (TET) are responsible for the separation of WS and CS samples and contribute positively to PC1. Peak 7 (FAN), selected as the reference peak, is located at zero on both *x* and *y* axes. These three peaks were found to be the characteristic peaks in differentiation between WS and CS samples, in the present study.

### 3.4 PCA of FIMS fingerprints

Typical FIMS fingerprint of WS6 and CS6 are shown in Figure 2a,b, respectively. It is impossible to discriminate 27 batches of samples which contain hundreds of variables intuitively. PCA scores plots obtained from the generated PCs is automation and simplification, moreover provides visual patterns and avoids subjective decisions.

**FIGURE 5:**
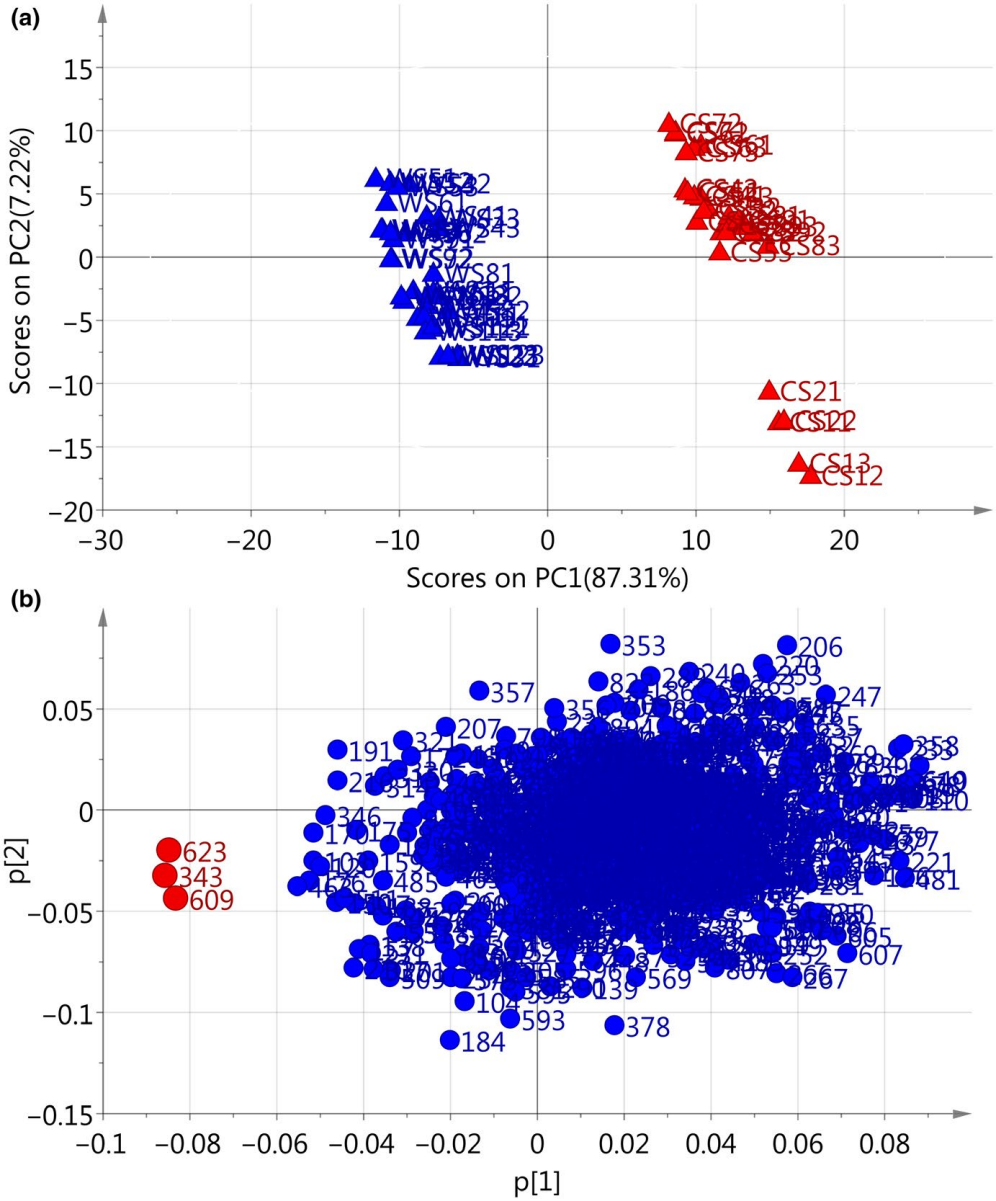
PCA scores plot (a) and loadings plot (b) for flow‐injection mass spectrometric fingerprints of wild and cultivated *Stephaniae tetrandrae* Radix samples

In present study, the variables were the absolute intensity values of the ions between *m/z* 100 and 1,000 (901 variables), and the observations were 81 samples. The most notable ions for WS are at *m/z* 175, 326, 333, 342, 343, and 623, and for CS are at *m/z* 118, 175, 326, 342, and 343. Figure 5a shows the PCA scores plot of FIMS fingerprints for the first two PCs. Intuitively, WS samples and CS samples are clearly separated from each other by PC1. WS samples are mainly clustered on the left in the negative PC1 area, with PC1 scores below zero. However, CS samples are gathered at the right side in the positive PC1 area, with PC1 scores above zero.

According to PCA loadings plot of FIMS fingerprints, the ions at *m/z* 609 (peak4, TET), 623 (peak7, FAN), and 343 (peak1, CYC) contribute negatively to PC1 (Figure 5). The effects of absolute intensity values of these ions decide the position of each sample. PCA loadings plot suggest that the ions at *m/z* 343, 609, and 623 were more important for differentiation between wild and cultivated *S. tetrandrae* samples. PCA from FIMS and HPLC‐UV fingerprints give us similar results. In summary, the ions at *m/z* 609 (peak4, TET), 623 (peak7, FAN), and 343 (peak1, CYC) were the most abundant peaks and played the most important role in differentiation between WS and CS samples in the present study.

Previous studies showed that TET is best characterized as a Ca^2+^‐entry blocker, current study indicated TET have more pharmacological effects, such as anti‐inflammatory, antimicrobial, antioxidant and antidiabetic (Bhagya & Chandrashekar, 2016) and anticancer (Li et al., 2019; Qiu et al., 2014) activities. FAN exhibits antioxidant, anti‐inflammatory (Liu et al., 2019) and against oxidative glutamate toxicity (Bao, Tao, & Zhang, 2019) effects. Especially, TET could increase blood sugar level from 0.5 to 5 hr after injection and returned to normal level 7 hr and decreased at 24 hr (Sun et al., 1992). These results reveal the complex regulation of glucose metabolism of TET; however, the mechanism remains poorly understood. Screening novel compounds from medical plant that effectively elevate glucose metabolism without producing side effects (Han, Wu, & Wang, 2019) is always a great challenge. The result of this study indicated that the wild *S. tetrandrae* radix contain high amount of these pharmacological active compounds that may be important in assessing the quality of the herb and related products.

## 4 CONCLUSIONS

High‐performance liquid chromatographic fingerprints provide more detailed information on chemical profiles of the samples, compared with FIMS fingerprints, but it requires longer analysis time (Ademiluyi, Aladeselu, Oboh, & Boligon, 2018). FIMS fingerprint is time saving and simple for fast screening. However, it is hard to analyze the data visually and intuitively (Zhao et al., 2015). With the aid of chemometrics, it provides visualized PCA scores plots and loadings plots for further analysis. Both fingerprinting techniques were used in the present study on discrimination between WS and CS samples. PCA scores plot displayed the differences of them, whereas loadings plot helped screening the markers which were critical variables (CYC, TET, and FAN in this study) for discrimination of the observations (WS and CS samples in this study). With the commercially available reference compounds, CYC, TET, and FAN were quantified. These three compounds are able to be used for quality control of *S. tetrandrae* radix and related products.

## CONFLICT OF INTEREST

The authors declare that they have no conflicts of interest.

## ETHICAL APPROVAL

This study does not involve any human or animal testing.
