# Supplementary figures and images for: Differentiation between wild and artificial cultivated Stephaniae tetrandrae radix using chromatographic and flow‐injection mass spectrometric fingerprints with the aid of principal component analysis
Source: Food Sci Nutr. 2020 Jun 23;8(8):4223–31. doi: 10.1002/fsn3.1717 (PMC7455950; doi:10.1002/fsn3.1717)

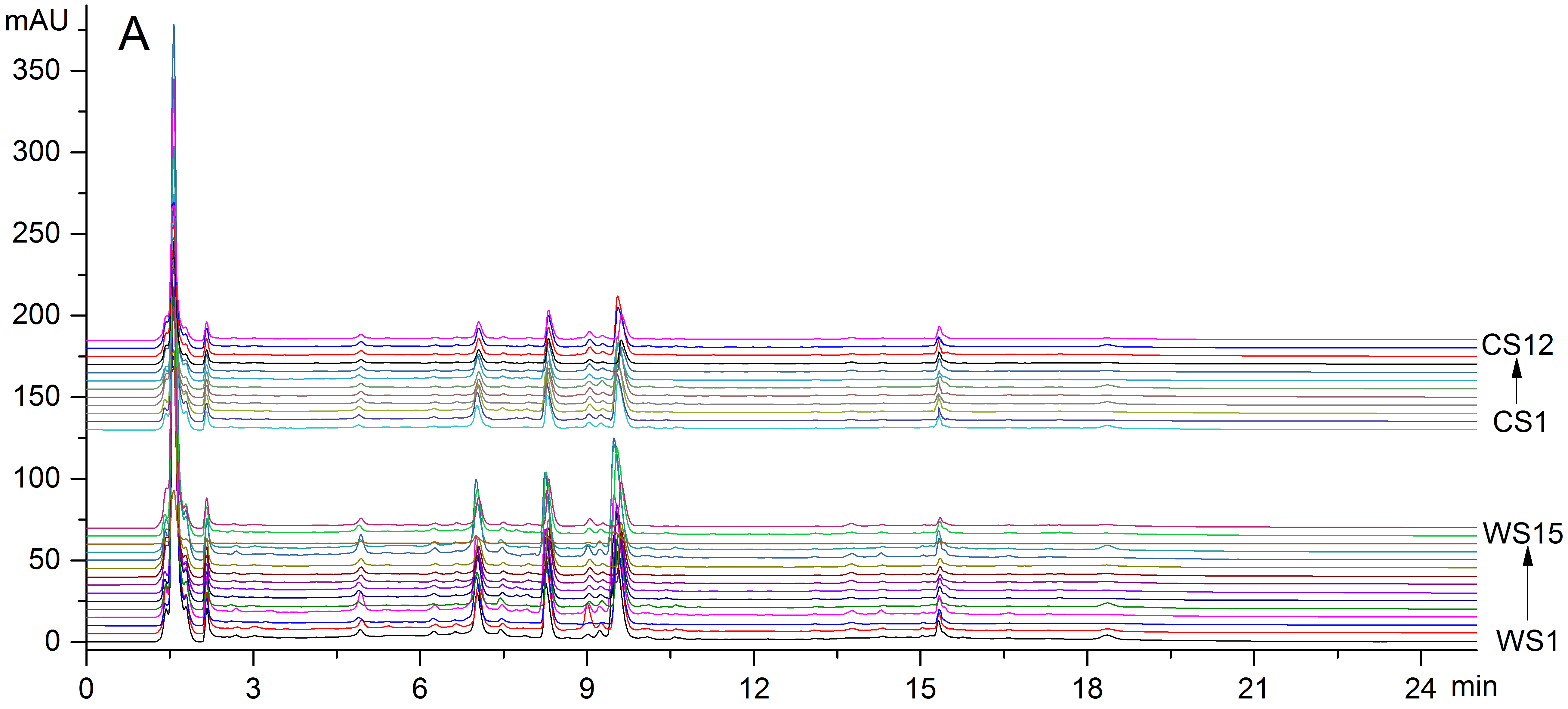

Supplement: Supplementary file 1 [file FSN3-8-4223-s001.jpg]

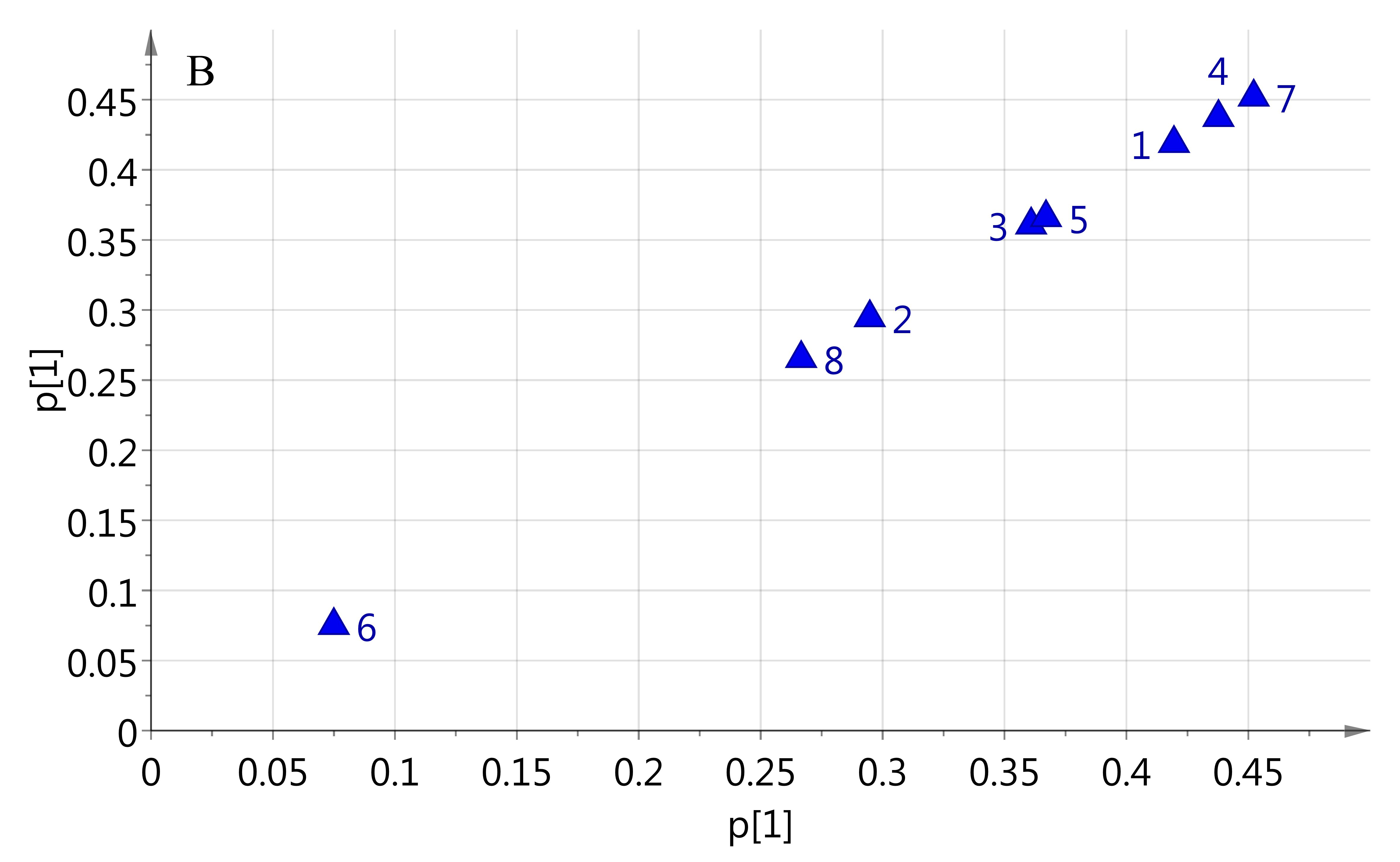

Supplement: Supplementary file 2 [file FSN3-8-4223-s002.jpg]
